# Hispidulin Inhibits Mast Cell-Mediated Allergic Inflammation through Down-Regulation of Histamine Release and Inflammatory Cytokines

**DOI:** 10.3390/molecules24112131

**Published:** 2019-06-05

**Authors:** Dong Eun Kim, Kyoung-jin Min, Min-Jong Kim, Sang-Hyun Kim, Taeg Kyu Kwon

**Affiliations:** 1 Department of Otolaryngology, School of Medicine, Keimyung University, 1095 Dalgubeoldaero, Dalseo-Gu, Daegu 42601, Korea; entkde@dsmc.or.kr; 2 Department of Immunology, School of Medicine, Keimyung University, 1095 Dalgubeoldaero, Dalseo-Gu, Daegu 42601, Korea; kyoungjin.min@gmail.com; 3 Department of Pharmacology, CMRI, School of Medicine, Kyungpook National University, Daegu 41944, Korea; wowdamien@hanmail.net (M.-J.K.); shkim72@knu.ac.kr (S.-H.K.)

**Keywords:** Hispidulin, mast cells, allergy, inflammation

## Abstract

Hispidulin (4′,5,7-trihydroxy-6-methoxyflavone) is a natural compound derived from traditional Chinese medicinal herbs, and it is known to have an anti-inflammatory effect. Here, we investigated the effect of hispidulin on the immunoglobulin E (IgE)-mediated allergic responses in rat basophilic leukemia (RBL)-2H3 mast cells. When RBL-2H3 cells were sensitized with anti-dinitrophenyl (anti-DNP) IgE and subsequently stimulated with DNP-human serum albumin (HSA), histamine and β-hexosaminidase were released from the cells by degranulation of activated mast cells. However, pretreatment with hispidulin before the stimulation of DNP-HSA markedly attenuated release of both in anti-DNP IgE-sensitized cells. Furthermore, we investigated whether hispidulin inhibits anti-DNP IgE and DNP-HSA-induced passive cutaneous anaphylaxis (PCA), as an animal model for Type I allergies. Hispidulin markedly decreased the PCA reaction and allergic edema of ears in mice. In addition, activated RBL-2H3 cells induced the expression of inflammatory cytokines (tumor necrosis factor-α and interleukin-4), which are critical for the pathogenesis of allergic disease, through the activation of c-Jun *N*-terminal kinase (JNK). Inhibition of JNK activation by hispidulin treatment reduced the induction of cytokine expression in the activated mast cells. Our results indicate that hispidulin might be a possible therapeutic candidate for allergic inflammatory diseases through the suppression of degranulation and inflammatory cytokines expression.

## 1. Introduction

Mast cells are one of the major immune cells, and are distributed in blood vessels, lymphoid organs, and external environment such as in skin, lungs, urogenital tracts and gastrointestinal tracts [1]. Mast cells protect our body from the damage by environmental dangers, and induce the inflammatory response [2]. Mast cells activated by various stimuli release histamines, proteoglycans, serotonin, and lipid mediators (prostaglandins and leukotrienes) [3]. These mediators are accumulated within granules and then are released into the extracellular environment by stimuli, resulting in the induction of inflammation and the recruitment of other immune cells, such as neutrophils, eosinophils, basophils, and monocyte/macrophages [4]. After that, mast cells synthesize and then secrete other pro-inflammatory cytokines (interleukins, GM-CSF, and TGF-β) and chemokines [5]. These inflammatory reactions by mast cells are closely related to allergic reactions. Upon exposure of an allergen, allergen-specific immunoglobulin E (IgE) bind to high-affinity IgE receptors (FcεRI) and crosslinking of adjacent IgE molecules induces aggregation of FcεRI, followed by degranulation in mast cells [6]. The multiple intracellular signaling pathways, protein kinase C and mitogen-activated protein kinases (MAPKs), are activated by the aggregation of Fc receptors, and activation of these signaling pathways modulate the inflammatory responses [4].

Hispidulin (4′,5,7-trihydroxy-6-methoxyflavone) is a natural compound derived from traditional Chinese medicinal herbs, such as *Artemisia vestita* [7] and *Arnica Montana L.* [8]. Several studies reported that hispidulin has multiple functions, including anti-fungal, anti-epileptic, anti-hypnotic, and anti-osteoclastogenesis activities [9,10,11]. Recently, anti-inflammatory effects of hispidulin have been reported. Hispidulin inhibits LPS-induced nitric oxide production and inflammatory mediators, such as inducible nitric oxide synthase, tumor necrosis factor (TNF)-α, and interleukin (IL)-1β in Raw264.7 and HT29 cells [12]. Hispidulin also inhibits kainic acid-induced production of proinflammatory cytokines [9]. However, the effect of hispidulin on allergic inflammation has not been elucidated. Therefore, our aim is to evaluate the effects of hispidulin on mast cell-mediated allergic inflammation and their underlying mechanism.

## 2. Results

### 2.1. Hispidulin Inhibits Mast Cell Degranulation

**Figure 1:**
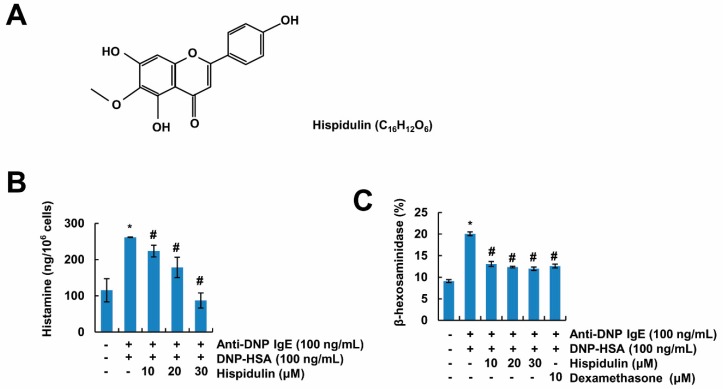
Hispidulin inhibits degranulation of mast cells. (**A**) The structure of hispidulin. (**B**,**C**) Anti-dinitrophenyl (DNP) immunoglobulin E (IgE) (100 ng/mL)-sensitized rat basophilic leukemia (RBL)-2H3 cells (sensitized overnight) were treated with hispidulin for 1 h, and then cells were stimulated with DNP-human serum albumin (HSA) (100 ng/mL) for 8 h (**B**) or 4 h (**C**). Histamine and β-hexosaminidase levels in culture supernatants of RBL-2H3 cells were detected using a fluorescence plate reader or a spectrophotometer, respectively. The values in **B** and **C** represent the means ± SEM from three independent experiments. * *p* < 0.01 compared to the control. ^#^ *p* < 0.01 compared to the anti-DNP IgE plus DNP-HSA.

Mast cells produce histamine, which is a key molecule in allergic responses. Therefore, inhibition of histamine release in mast cells is a useful therapeutic target for the treatment of allergic symptoms. Since rat basophilic leukemia (RBL)-2H3 cells are suitable cells to examine the effects of mast cell-mediated inflammation [13], we used these cells to investigate the anti-allergic effects of hispidulin (Figure 1A). As shown in Figure 1B, stimulation by dinitrophenyl (DNP)-human serum albumin (HSA) induced the release of histamine in anti-DNP-IgE sensitized cells. However, hispidulin markedly inhibited histamine release in a concentration-dependent manner in activated cells. Furthermore, β-hexosaminidase, which is localized in the granules of mast cells, was also released by the stimulation with DNP-HSA (Figure 1C). Hispidulin also inhibited β-hexosaminidase release, and this inhibitory effect was similar with that of dexamethasone, the positive control drug (Figure 1C). Therefore, these data indicate that hispidulin inhibits degranulation of mast cells.

### 2.2. Hispidulin Reduces IgE-Mediated Local Cutaneous Anaphylaxis Reaction

**Figure 2:**
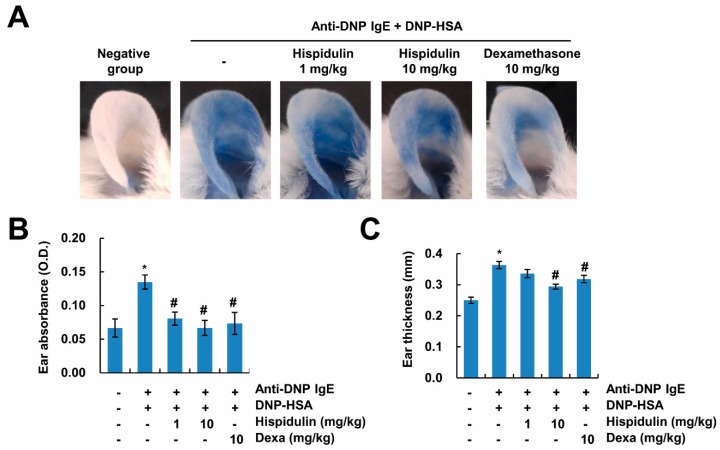
Hispidulin attenuates passive cutaneous anaphylaxis. (**A**) The ear skin of mice (*n* = 5/group) was sensitized with an intradermal injection of anti-DNP IgE (0.5 μg/site) for 48 h. Hispidulin was intraperitoneally administered at doses of 1 and 10 mg/kg body weight (BW) 1 h before the intravenous injection of a DNP-HSA and 4% Evans blue (1:1) mixture. Thirty minutes later, the ears were collected to measure the dye pigmentation, and the thickness of both ears was measured. The dye was extracted as described in the Materials and Methods section and detected using a spectrophotometer (**B**). Ear thickness was measured with a dial thickness gauge (**C**). The values in B and C represent the means ± SEM from five determinations. * *p* < 0.01 compared to the control. ^#^ *p* < 0.05 compared to the anti-DNP IgE plus DNP-HSA.

To examine the effects of hispidulin on the IgE-mediated allergic reaction in vivo, we used a passive cutaneous anaphylaxis (PCA) model. PCA is used in animal models for immediate-type allergic reactions. After challenges of the antigen, histamine secreted by mast cells increases vascular permeability, causing the appearance of blue spots by Evans blue. Therefore, the PCA reaction is detected by the amount of Evans blue dye extravasation, depending on vascular permeability. As shown in Figure 2A,B, extravasation of Evans blue dyes was markedly detected, and administration of hispidulin reduced the PCA reaction. In addition, increased vascular permeability induced the thickening of the ear, and this phenomenon was also inhibited by hispidulin (Figure 2C). These data indicate that hispidulin attenuates the IgE-mediated passive cutaneous anaphylaxis reaction.

### 2.3. Hispidulin Inhibits Expression of Inflammatory Cytokines

**Figure 3:**
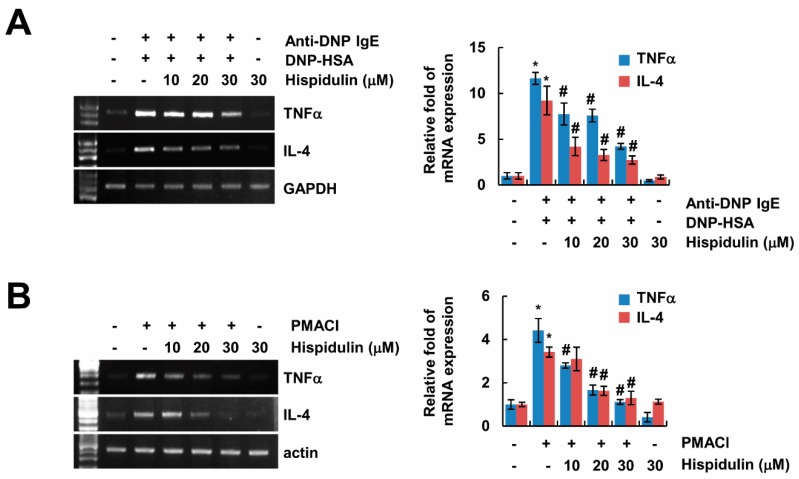
Hispidulin inhibits expression of inflammatory cytokines. (**A**) Anti-DNP IgE (100 ng/mL)-sensitized RBL-2H3 cells were pretreated with hispidulin for 1 h, and then cells were stimulated with DNP-HSA (100 ng/mL) for 3 h. Bar graph represents means ± SEM of tumor necrosis factor (TNF)-α and interleukin (IL)-4 relative intensities from three independent experiments. (**B**) Human mast cell (HMC)-1 cells were pretreated with hispidulin for 1h, and then cells were treated with 40 nM phorbol 12-mystate 13-acetate (PMA) plus 1 μg/mL A23187 for 3 h. The mRNA expression was determined by RT-PCR. Bar graph represents means ± SEM of TNF-α and IL-4 relative intensities from three independent experiments. The band intensity was measured using ImageJ. * *p* < 0.01 compared to the control. ^#^ *p* < 0.05 compared to the anti-DNP IgE plus DNP-HSA or PMA plus calcium ionophore A23187 (PMACI).

Next, we investigated whether hispidulin inhibits expression of pro-inflammatory cytokines, which are related with the pathogenesis of allergic disease. Sensitized RBL-2H3 cells increased expression of TNF-α and IL-4 by DNP-HSA stimulation, and hispidulin inhibited expression of them in a concentration-dependent manner (Figure 3A). Furthermore, we also checked the effect of hispidulin in human mast cell lines (HMC-1). When HMC-1 cells were treated with phorbol 12-mystate 13-acetate (PMA) plus calcium ionophore A23187 (PMACI), expression of TNF-α and IL-4 was markedly increased, and hispidulin attenuated the expression of both (Figure 3B). These data indicate that hispidulin inhibits inflammatory cytokine expression in activated mast cells.

### 2.4. Hispidulin Inhibits Expression of Inflammatory Cytokines via Inhibition of JNK Phosphorylation

**Figure 4:**
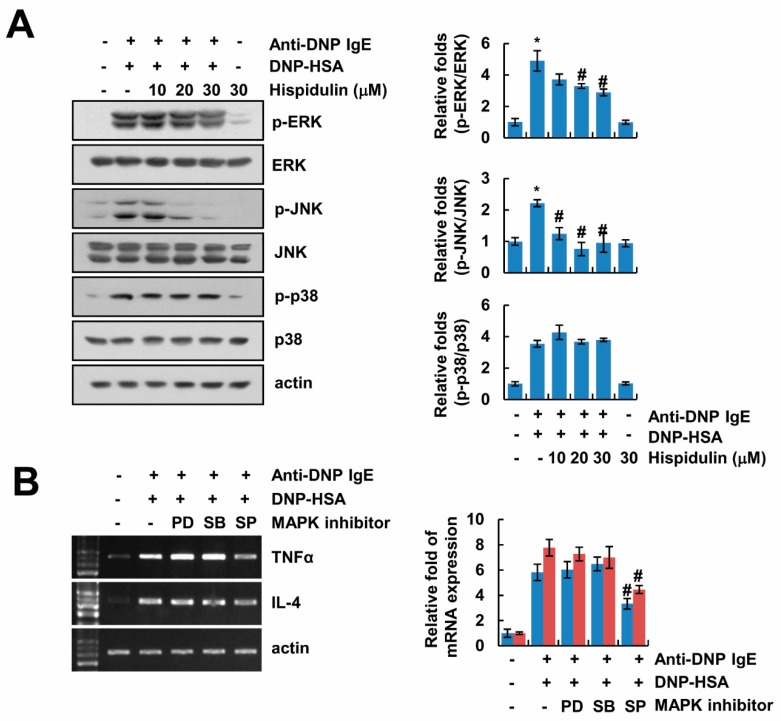
Inhibition of JNK mitogen-activated protein kinases (MAPK) by hispidulin is involved in down-regulation of inflammatory cytokines expression. (**A**) Anti-DNP IgE (100 ng/mL)-sensitized RBL-2H3 cells were pretreated with hispidulin for 1 h, and then cells were stimulated with DNP-HSA (100 ng/mL) for 30 min. The expression and phosphorylation of protein was determined by Western blot. Bar graph represents means ± SEM of *p*-ERK/ERK, *p*-p38/p38, and *p*-JNK/JNK relative intensities from three independent experiments. (**B**) Anti-DNP IgE (100 ng/mL)-sensitized RBL-2H3 cells were pretreated with ERK inhibitor (50 μM PD98059;PD), p38 MAPK inhibitor (10 μM SB203580;SB), and JNK inhibitor (10 μM SP600125;SP) for 1 h, and then cells stimulated with DNP-HSA (100 ng/mL) for 3 h. The mRNA expression was determined by RT-PCR. Bar graph represents means ± SEM of TNF-α and IL-4 relative intensities from three independent experiments. The band intensity was measured using ImageJ. * *p* < 0.01 compared to the control. ^#^ *p* < 0.05 compared to the anti-DNP IgE plus DNP-HSA.

We investigated how hispidulin inhibits TNF-α and IL-4 expression in activated mast cells. Previous studies reported that activation of MAPK is one of the important signals in the expression of inflammatory cytokines in mast cells [4]. Therefore, we examined the effects of hispidulin on MAPK activation. Hispidulin slightly inhibited anti-DNP-IgE/DNP-HSA-induced phosphorylation of ERK, and markedly blocked phosphorylation of JNK (Figure 4A). In contrast, phosphorylation of p38 MAPK was not altered by hispidulin treatment (Figure 4A). Furthermore, we found that the JNK inhibitor (SP600125) reduced anti-DNP-IgE/DNP-HSA-mediated up-regulation of TNF-α and IL-4 expression (Figure 4B). These data indicate that hispidulin inhibits JNK activation, resulting in down-regulation of inflammatory cytokines expression in anti-DNP-IgE/DNP-HSA-treated mast cells.

## 3. Discussion

In our study, we showed that hispidulin attenuated the allergic inflammatory response. Hispidulin inhibited the release of histamine and β-hexosaminidase in activated mast cells and attenuated IgE-mediated passive cutaneous anaphylaxis in mouse allergy models. The anti-allergic effects of hispidulin were related with the down-regulation of inflammatory cytokines expression, and inhibition of JNK MAPK phosphorylation by hispidulin was critical for inhibition of inflammatory cytokines expression in activated mast cells. Our results suggest that hispidulin is a possible therapeutic candidate for therapy of allergic disorders.

Hispidulin is a polyphenolic flavonoid possessing multiple functions. First, hispidulin has anti-cancer effects. Hispidulin induces apoptosis in human renal carcinoma [14], human hepatocellular carcinoma [15], glioblastoma [16], and acute myeloid leukemia [17]. In addition, hispidulin inhibits epithelial-mesenchymal transitions, which are important for the initiation of cancer metastasis in human colon carcinoma cells [18], and suppresses metastasis in renal cell carcinoma [19]. Second, hispidulin modulates lipid metabolism. Hispidulin directly binds to PPAR-α, and then modulates expression of lipid metabolism enzymes (fatty acid binding protein 1/2, and long chain acyl-CoA synthetase 1) [20]. Recently, it was also reported that hispidulin inhibits adipogenesis via inhibition of PPAR-γ expression [21]. Third, hispidulin reduces glutamate toxicity. Hispidulin inhibits glutamate release from rat cerebrocortical nerve terminals [22], and along with this effect, hispidulin reduces kainic acid-induced neurotoxicity [9]. Finally, hispidulin has anti-inflammatory effects. Hispidulin inhibits nitric oxide production and expression of inflammatory cytokines in lipopolysaccharide-treated Raw264.7 macrophage cells [12]. Furthermore, Akram et al. reported that hispidulin induces Nrf2 nuclear translocation and up-regulates hemeoxygenase-1 expression [23]. In this study, we found anti-inflammatory and anti-allergic functions of hispidulin. Therefore, we examined the effects of hispidulin on phosphorylation of Nrf2. Phosphorylation of Nrf2 was not increased by hispidulin treatment in activated mast cells, and hispidulin alone also had no effect on phosphorylation of Nrf2 (negative data not shown). Anti-inflammatory function of hispidulin might be independent of Nrf2 activation in our condition. To investigate the anti-allergic effects of natural compounds, RBL-2H3 cells are commonly used as a mast cell model. Anti-DNP IgE-primed and DNP-HSA-stimulated mast cells induce allergic responses (induction of histamine and β-hexosaminidase release and Th2 cytokines expression). However, these responses do not occur in the absence of sensitization with anti-DNP IgE or in the absence of stimulation with DNP-HSA [24,25,26,27]. Therefore, we performed our experiments in the condition of their presence with anti-DNP IgE sensitization and DNP-HSA stimulation. We found that hispidulin markedly inhibited degranulation and expression of inflammatory cytokines in activated mast cells, and attenuated vascular permeability and edema in PCA models (Figure 1, Figure 2 and Figure 3).

Recently, a variety of natural compounds as new drug candidates have been reported to reduce mast cell-mediated allergic inflammation. For example, nothofagin, which is the dihydrochalcone of unprocessed rooibos tea, inhibits mast cell-mediated cytokine production and allergic responses [28]. Nothofagin inhibits activation of the Src family of kinases (Lyn and Syk), Akt, and the nuclear translocation of nuclear factor (NF)-κB by aggregation of antigen–IgE complexes bound to FcεRI, resulting in down-regulation of TNF-α, IL-4, and IL-6 expression [28]. Elaeocarpusin isolated from *Elaeocarpus sylvestris* L. also inhibits mast cell-mediated allergic responses via inhibition of Lyn, Syk, and NF-κB signaling [29]. *Davallia mariesii* Moore (Drynaria rhizome) inhibits COX-2 expression and PGD_2_ secretion in anti-DNP IgE/DNP-HSA-activated mast cells [30]. *Davallia mariesii* Moore inhibits phosphorylation of ERK MAPK, as well as Lyn, Syk, and Akt [30]. Anti-DNP IgE/DNP-HSA activated MAPK signaling pathways in RBL-2H3 cells (Figure 4A), but hispidulin inhibited phosphorylation of ERK and JNK in activated mast cells (Figure 4A). Among them, only the JNK inhibitor reduced expression of TNF-α and IL-4 (Figure 4B). Therefore, inhibition of JNK activation by hispidulin might be important for the reduction of mast cells activation. Inhibition of mast cells activation is an important strategy in controlling the outcome of allergic responses and disorders.

## 4. Materials and Methods

### 4.1. Reagents and Cell Culture

Anti-DNP IgE, DNP-human serum albumin (HSA), phorbol 12-mystate 13-acetate (PMA), A23187, dexamethasone and *o*-phthaldialdehyde were purchased from Sigma (St. Louis, MO, USA). Hispidulin was purchased from Santa Cruz Biotechnology (Santa Cruz, CA, USA). Human mast cells (HMC)-1 and RBL-2H3 cells were grown in IMDM (GIBCO, Grand Island, NY, USA) and Dulbecco’s modified Eagle’s medium (GIBCO), respectively, and supplemented with 100 units/mL penicillin/streptomycin, and heat-inactivated 10% fetal bovine serum (GIBCO) in 5% CO_2_ at 37 °C. RBL-2H3 cells were used throughout the study at a passage number ranging from 4 to 8. The anti-ERK, anti-phospho-ERK, anti-phospho-JNK, anti-JNK, anti-phospho-p38 MAPK, and anti-p38 MAPK antibodies were purchased from Cell Signaling Technology (Beverly, MA, USA). The anti-ERK antibody was obtained from Transduction Laboratories (Lexington, KY, USA). The actin antibody was purchased from Sigma (St. Louis, MO, USA).

### 4.2. Histamine Release

Histamine contents in cultured cell supernatants were measured by the *o*-phthaldialdehyde spectrofluorometric procedure as previously described [31]. Anti-DNP IgE (100  ng/mL)-sensitized RBL-2H3 cells were pre-incubated with hispidulin at 37 °C for 1 h, and then stimulated with DNP-HSA for 8 h. The fluorescent intensity was measured at an emission of 438 nm and excitation of 353 nm using a spectrofluorometer.

### 4.3. β-Hexosaminidase Release

Anti-DNP IgE (100  ng/mL)-sensitized RBL-2H3 cells were washed three times in phosphate-buffered saline (PBS), treated with hispidulin for 1  h, and then stimulated with DNP-HSA (100  ng/mL) for 4  h. After incubation, the cells were separated from the media by centrifugation at 150  g for 5  min at 4  °C; subsequently, 40  μL of the supernatant or the cell pellet after lysis with 0.5% Triton X-100 was transferred to a 96-well plate and incubated at 37  °C with 40  μL of 0.1  M citrate buffer (pH 4.5) containing 1  mM *p*-nitrophenyl-*N*-acetyl-β-d-glucosaminide. After 1 h, the reaction was stopped with 200 μL of 0.1 M Na_2_CO_3_/NaHCO_3_. The absorbance was measured with a spectrophotometer (Molecular Devices) at 405 nm. The percentage β-hexosaminidase release was calculated from the equation: [β-hexosaminidase release (%) = (absorbance of supernatant)/(absorbance of supernatant + absorbance of pellet) × 100].

### 4.4. Animals

The male ICR mice (6 weeks) were purchased from Dae-Han Experimental Animal Center (Daejeon, Korea). All the mice were allowed 1 week to acclimatize to the surroundings before the experiments, and were kept at 25 ± 2 °C, with a relative humidity of 55 ± 5% and a 12 h light–dark cycle. The study protocol was approved by the IRB Keimyung University Ethics Committee (KM-2018-15).

### 4.5. Passive Cutaneous Anaphylaxis (PCA)

An IgE-dependent cutaneous reaction was carried out as described previously [32]. The mice were injected intradermally into the ear with 0.5 μg of anti-DNP IgE. After 48 h, each mouse received an injection of 1 μg of DNP-HSA containing 4% Evans blue (1:4) via the tail vein. Hispidulin were intraperitoneally administered at doses 1 and 10 mg/kg body weight (BW) 1 h before the challenge. Thirty minutes after the challenge, the mice were killed and the ears were removed for measurement of the pigment area. The amount of dye was determined colorimetrically after extraction with 1 mL of 1 M KOH and 9 mL of a mixture of acetone and phosphoric acid (5:13). The intensity of absorbance (100 µL) was measured at 620 nm in a spectrophotometer (UV-1201; Shimadzu, Kyoto, Japan).

### 4.6. Reverse Transcription Polymerase Chain Reaction (RT-PCR)

Total RNA was isolated using the TriZol reagent (Life Technologies, Gaithersburg, MD, USA), and cDNA was prepared using M-MLV reverse transcriptase (Gibco-BRL, Gaithersburg, MD, USA) according to the manufacturer’s instructions. The following primers were used for the amplification of rat TNF-α, rat IL-4, rat GAPDH, human TNF-α, human IL-4, and human actin: rat TNF-α (forward) 5′-CAT CTG CTG GTA CCA CCA GTT-3′ and (reverse) 5′-TGA GCA CGA AAA GCA TGA TC-3′; rat IL-4 (forward) 5′-ACC TTG CTG TCA CCC TGT TC-3′ and (reverse) 5′-TTG TGA GCG TGG ACT CAT TC-3′; rat GAPDH (forward) 5’-TCC CTC AAG ATT GTC AGC A-3’ and (reverse) 5’-AGA TCC ACA ACG GAT ACA TT-3’; human TNF-α (forward) 5′-CCT ACC AGA CCA AGG TCA AC-3′ and (reverse) 5′-AGG GGG TAA TAA AGG GAT TG-3′; human IL-4 (forward) 5′-ATG GGT CTC ACC TCC CAA CTG CT-3′ and (reverse) 5′-CAG CTC GAA CAC TTT GAA TAT TTC TCT CTC-3′; and actin (forward) 5′-GGC ATC GTC ACC AAC TGG GAC-3′ and (reverse) 5′-CGA TTT CCC GCT CGG CCG TGG-3′. The PCR amplification was carried out using the following cycling conditions: 94 °C for 3 min followed by 17 (actin) or 25 cycles (others) of 94 °C for 40 s, 56 °C for 40 s, 72 °C for 1 min, and a final extension at 72 °C for 5 min. The amplified products were separated by electrophoresis on a 1.5% agarose gel and detected under UV light.

### 4.7. Western Blot Analysis

Cells were washed with cold PBS and lysed on ice in 50 μL of lysis buffer (50 mM Tris–HCl, 1 mM EGTA, 1% Triton X-100, 1 mM phenylmethylsulfonyl fluoride, pH 7.5). Lysates were centrifuged at 10,000 g for 15 min at 4 °C, and the supernatant fractions were collected. Proteins were separated by SDS–PAGE and transferred to an Immobilon-P membrane [33]. Specific proteins were detected using an enhanced chemiluminescence (ECL) Western blot kit according to the manufacturer’s instructions.

### 4.8. Statistical Analysis

Using a small a sample size makes it difficult to produce meaningful and reproducible results, and too large a sample size makes it difficult to determine qualitatively good inductive conclusions. The sample size (three independent experiments) used in this study was decided by experimental experience and previous papers, and the results were tested for significance. Statistical analyses were performed using SAS statistical software (SAS Institute, Cary, NC, USA). Treatment effects were analyzed using analysis of variance, followed by Duncan’s multiple range tests. The value of *p* < 0.05 was used to indicate significance.

## 5. Conclusions

Collectively, our findings provide evidence and a molecular mechanism for the contribution of hispidulin in the inhibition of mast cell-mediated immediate-type hypersensitivity. Therefore, we propose that hispidulin may be a candidate as a therapeutic drug to treat the allergic response.

## Abbreviations

TNF	Tumor necrosis factor
IL	Interleukin
HAS	Human serum albumin
PCA	Passive cutaneous anaphylaxis
MAPK	Mitogen-activated protein kinase
